# The short international physical activity questionnaire: cross-cultural adaptation, validation and reliability of the Hausa language version in Nigeria

**DOI:** 10.1186/1471-2288-11-156

**Published:** 2011-11-22

**Authors:** Adewale L Oyeyemi, Adetoyeje Y Oyeyemi, Babatunde O Adegoke, Fatima O Oyetoke, Habeeb N Aliyu, Salamatu U Aliyu, Adamu A Rufai

**Affiliations:** 1 Department of Physiotherapy, College of Medical Sciences, University of Maiduguri, Nigeria; 2 Department of Physiotherapy, College of Medicine, University of Ibadan, Nigeria

## Abstract

**Background:**

Accurate assessment of physical activity is important in determining the risk for chronic diseases such as cardiovascular disease, stroke, type 2 diabetes, cancer and obesity. The absence of culturally relevant measures in indigenous languages could pose challenges to epidemiological studies on physical activity in developing countries. The purpose of this study was to translate and cross-culturally adapt the Short International Physical Activity Questionnaire (IPAQ-SF) to the Hausa language, and to evaluate the validity and reliability of the Hausa version of IPAQ-SF in Nigeria.

**Methods:**

The English IPAQ-SF was translated into the Hausa language, synthesized, back translated, and subsequently subjected to expert committee review and pre-testing. The final product (Hausa IPAQ-SF) was tested in a cross-sectional study for concurrent (correlation with the English version) and construct validity, and test-retest reliability in a sample of 102 apparently healthy adults.

**Results:**

The Hausa IPAQ-SF has good concurrent validity with Spearman correlation coefficients (ρ) ranging from 0.78 for vigorous activity (Min Week^-1^) to 0.92 for total physical activity (Metabolic Equivalent of Task [MET]-Min Week^-1^), but poor construct validity, with cardiorespiratory fitness (ρ = 0.21, p = 0.01) and body mass index (ρ = 0.22, p = 0.04) significantly correlated with only moderate activity and sitting time (Min Week^-1^), respectively. Reliability was good for vigorous (ICC = 0.73, 95% C.I = 0.55-0.84) and total physical activity (ICC = 0.61, 95% C.I = 0.47-0.72), but fair for moderate activity (ICC = 0.33, 95% C.I = 0.12-0.51), and few meaningful differences were found in the gender and socioeconomic status specific analyses.

**Conclusions:**

The Hausa IPAQ-SF has acceptable concurrent validity and test-retest reliability for vigorous-intensity activity, walking, sitting and total physical activity, but demonstrated only fair construct validity for moderate and sitting activities. The Hausa IPAQ-SF can be used for physical activity measurements in Nigeria, but further construct validity testing with objective measures such as an accelerometer is needed.

## Background

There is a wide consensus on the benefits of physical activity in health and in disease [1]. Accurate assessment of physical activity is important in determining the risk for cardiovascular disease, stroke, type 2 diabetes, cancer and obesity in developing countries [2,3]. One viable strategy for stemming the rising incidence of chronic diseases in developing countries is to increase the population's level of physical activity [3,4], but epidemiological studies on physical activity remain sparse in sub-Saharan Africa, due to the lack of culturally relevant physical activity tools in indigenous African languages and an appropriate system for monitoring [5].

The International Physical Activity Questionnaire (IPAQ) was developed as an instrument for cross-national assessment of physical activity and for standardizing measures of health-related physical activity behaviors of population in many countries and in different socio-cultural contexts [6,7]. Both the long (31-item) and the short (7-item) versions of this instrument, which elicits information on physical activity over the 'last seven-day' period, have been used to compare physical activity behaviors among and between populations [6,8-10]. The short form of IPAQ (IPAQ-SF) has been recommended for population prevalence studies, where time is limited, because it is easier and more feasible to complete than the long form [6].

The IPAQ has been translated into many languages and extensively tested in many countries around the world, except in sub-Saharan Africa [7,11-15]. In order to make the IPAQ applicable for research among non-English speaking populations in sub-Saharan Africa, it needs to be translated, culturally adapted and properly evaluated for psychometric properties. In sub-Saharan Africa where the sociocultural and physical environments are distinct from those of other parts of the world [16], mere translation of the IPAQ may be insufficient to maintain content validity, as important cultural differences can produce a translated version that lacks experiential and conceptual equivalence [17].

Culturally adapting the IPAQ, rather than developing a new physical activity questionnaire, is economical and it may facilitate future comparisons among populations. In West Africa, Hausa is a widely spoken language with over 50 million native speakers and 15 million non-native speakers in Northern Nigeria, the Republic of Niger, Northern Cameroon and Ghana [18]. The purpose of this study was to translate and cross-culturally adapt the English IPAQ-SF, and to evaluate aspects of the validity and reliability of the Hausa-translated and culturally adapted version of the IPAQ-SF.

## Methods

### Setting

Maiduguri is the largest city and the capital city in the state of Borno. This state is located in North-Eastern Nigeria, and has an estimated population of 4, 151, 193 people, consisting of Kanuri, Shuwa Arabs, Hausa, Fulfude and other ethnic groups [19,20]. The state covers an area of 72, 609 sq kilometer with a population density of 57 people/square kilometer, and attracts immigrants from the Republic of Cameroon, the Republic of Niger and the Chad Republic [20,21]. The diverse inhabitants of Maiduguri predominantly use the Hausa language as the common means of communication and commercial activities [22].

### The International Physical Activity Questionnaire

The questionnaire collects information on the time (i.e., number of days and average time per day) spent being physically active and measures vigorous-intensity activity, moderate-intensity activity, walking activity, and sitting in the last seven consecutive day period. These activity categories may be treated separately to obtain the specific activity patterns or multiplied by their estimated value in Metabolic Equivalent of Tasks (METs) and summed to gain an overall estimate of physical activity in a week http://www.ipaq.ki.se. One MET represents the energy expended while sitting quietly at rest and is equivalent to 3.5 ml/kg/min of VO_2 _Max [23]. The MET intensity values used to score IPAQ questions in this study were vigorous (8 METs), moderate (4 METs) and walking (3.3 METs) http://www.ipaq.ki.se. The IPAQ sitting question is an indicator of the time spent in sedentary activity and was not included as part of the summary score of total physical activity. Data were cleaned to ensure that the daily time spent on each of vigorous, moderate and walking activities ranged between 10 and 180 minutes for all participants http://www.ipaq.ki.se.

The outcomes measures used in the present study were (1) minutes reported in vigorous, moderate, walking and sedentary activities per week (Min week^-1^) and (2) MET minutes per week. Time spent in each activity category was derived by multiplying the number of days per week with the minutes spent doing the activity per day, while total weekly physical activity (MET-Min week^-1^) was calculated by multiplying the number of minutes spent in each activity category with the specific MET score for each activity.

### Procedures

To establish good face and content validity, the translation and cultural adaptation of the IPAQ-SF was performed in several steps following the guidelines prescribed by the IPAQ core group http://www.ipaq.ki.se. The English version of IPAQ-SF was translated into the Hausa language by two independent translators, both native speakers of Hausa who speak, read and write Hausa as well as speak, read and write English. One of the translators was a Hausa language expert based in the Linguistic Department of the University of Maiduguri, and the second translator was a physiotherapist clinician at a teaching hospital who was knowledgeable about the concept of cardiorespiratory fitness examined in IPAQ. The two translations, one each from the translators, were synthesized into a single Hausa version by a panel of experts consisting of the translators, two physiotherapists who specialize in exercise physiology and sports physiotherapy, the principal investigator, and a university student who was raised in Hausa language and culture. Following back translations of the synthesized Hausa version by two university lecturers from the Hausa ethnic group in Nigeria, each of them with over seven years of professional experience in health sciences, the panel of experts compared the two back translations with the original English version to ensure that the concept measured by IPAQ had not been lost during the translations.

The synthesized translation and the two back translations were then merged into one pre-final version of the Hausa IPAQ-Short Form (Hausa IPAQ-SF). The expert panel then compared the original English IPAQ and the pre-final Hausa version for conceptual equivalence (conceptual meaning to terms and concept in the Hausa population), experiential equivalence (cultural relevance of the tasks and examples used in the questionnaire), linguistic equivalence (meaning and grammatical correctness), and metric equivalence (ensuring that the substituting cultural items and examples of activity are equivalent in intensity with the original items and examples). The compendium of physical activity (available at http://prevention.sph.sc.edu) was used by the expert panel to ensure that the substitute culturally acceptable examples and items were equivalent in MET to the original examples and items in the English version.

The pre-final version of the Hausa IPAQ-SF was self-administered to 12 Hausa-speaking and Hausa-writing volunteers from a broad range of backgrounds. After the completion of the survey, each of the volunteers was separately interviewed by the principal investigator for their understanding of the words in the questionnaire, the clarity of each item, and their opinion and suggestions for improvement. They were also asked to indicate if any question made them feel uncomfortable and if any relevant items were not included in the questionnaire. Items that were difficult to understand as expressed by two or more volunteers were referred to the expert panel for consideration and the panel's recommendations were incorporated in all such cases.

### Evaluation of Psychometric Properties

The final version of the Hausa IPAQ-SF (Additional file 1) was evaluated for validity and reliability in a non-probability sample of 102 apparently healthy individuals, age 20-65 years. Participants for the psychometric study were recruited directly from various workplaces (e.g., university, teaching hospital, private establishments) and neighborhoods in Maiduguri city. Demographic characteristics including educational level and employment status of the participants were obtained on their first day of contact for the study. Height and weight were measured using standardized equipment. Body mass index (BMI) was calculated as body weight divided by the square of height (kg m^-2^). Participants were eligible for this study if they were willing to complete surveys in both English and Hausa languages and were not having any disability that prevented independent walking. One of the researchers (F.O.O) was in attendance to provide translation and interpretation assistance to participants (n = 7) who were unable to independently complete the survey.

Concurrent validity of the Hausa IPAQ-SF was assessed by comparing the durations (Min week^-1^) of physical activity (vigorous, moderate, walking and sitting) from the Hausa IPAQ-SF with that obtained from the original English version of IPAQ-SF. A simple random technique (flip of coin) was used to determine the order of administration of the two questionnaires. Participants whose coin returned with heads completed the Hausa IPAQ-SF first, while those with tails completed the original English version first. An interval of one hour was allowed between administrations of the two questionnaires. Craig et al. [6], in a 12-country international study on validity and reliability of IPAQ, directly compared different versions of IPAQ and described the process as concurrent or inter method validation.

For construct validity, the participants' rate pressure product (RPP) was compared with duration (Min week^-1^) of physical activity (vigorous, moderate, walking and sitting) from the Hausa IPAQ-SF. Rate pressure product (RPP) is an index of cardiorespiratory fitness and was derived by multiplying participants' resting systolic blood pressure and heart rate [23]. The systolic blood pressure and heart rate were measured with the Dinamap (model 8100/8101) digital blood pressure measuring device. Three measurements were taken at intervals of 3-5 minutes, and the mean systolic blood pressure and heart rate were used for computing the RPP used in the analysis. A correlation between duration in physical activity and RPP would suggest that the Hausa IPAQ-SF was sensitive to construct of physical activity such as cardiorespiratory fitness. Other important construct validity measures, such as VO_2 _max, motion sensors, diaries and indicators of lipid and glucose metabolism [12-14], were not used in this study due to cost and ease of utility.

Test-retest reliability was completed by administering the Hausa IPAQ-SF twice within a 1-week time frame. Participants during the retest administration were asked to focus on the 7-day physical activity recalled during the first testing. The 7-day time interval was chosen because IPAQ asks about activity in the last 7 days. All participants provided informed consent and the Research Ethical Committee of the University of Maiduguri Teaching Hospital, Nigeria gave approval for the protocol of the study before its commencement.

### Statistical Analysis

Descriptive data were reported as mean, standard deviation and percentages. Mean group differences in physical activity (Min week^-1^) by gender and socioeconomic status were examined by independent t-test. To assess validity, the non-parametric Spearman correlation coefficients (ρ) were calculated to assess the relationship between Min week^-1 ^of physical activity from the Hausa IPAQ-SF and rate pressure product and body mass index for construct validity, and with Min week^-1 ^in physical activity from the English version of IPAQ for concurrent validity. Pearson correlation coefficients were also used for comparisons, but we only present results from the Spearman correlation analyses, because our results and conclusions were similar. The two-way mixed model (single measure) intraclass correlation coefficient (ICC) was calculated to evaluate the test-retest reliability. A 95% confidence interval was used to describe the variety/differences in the ICCs by gender and socioeconomic status. Percent agreement was not reported because it overestimated the reliability estimates of our data. To prevent type 2-error due to the small sample size in the socioeconomic status-based analyses, education and employment status were respectively grouped into two categories: high school education or no high school education, and employed or not employed (homemaker, student, retired, or unable to work). The agreement levels ratings of poor (0-0.2), fair (0.2-0.4), moderate (0.4-0.6), substantial (0.6-0.8) and almost perfect (0.8-1.0), suggested by Landis and Koch, were used when interpreting the reliability results [24].

The Bland-Altman analysis was used to provide an indication of the heteroscedasticity of the data, and 95% limits of agreement were used for describing the total error between the Hausa IPAQ-SF and the original English IPAQ, and the retest Hausa IPAQ. Variables used for the Bland-Altman analysis were weekly time spent in moderate activity according to the Hausa IPAQ-SF versus the original English IPAQ, and weekly time in moderate activity on first administration of Hausa IPAQ-SF versus the retest administration. Data were entered and analyzed using Statistical Package for the Social Science (SPSS), version 15.0 for windows (SPSS Inc., Chicago, Illinois, USA) and the level of significance was set at p < 0.05.

## Results

The discrepancies observed during the translation and cultural adaptation of English IPAQ-SF into Hausa are presented in Table 1. For the test of the pre-final version of Hausa IPAQ-SF, 7 participants out of the 12 surveyed were available for interview after they completed the questionnaire (4 men, 3 women; mean age 39.4 years; age range 31-53 years). During the testing of the pre-final version, two items were changed because they raised confusion among the participants. For example, the word '*aikin karfi'*, an Hausa translation of physical activity, can be misunderstood as referring to being restless, so adding the phrase 'physical exercise' (i.e., *motsa jiki*) was needed to clarify that it was referring to a health behavior. Walking to travel from place to place was originally translated to '*tafiya daga wannan wuri zuwa wancan*', but was frequently understood by almost every participant as walking to travel out of town. It was finally translated to '*tafiya (tattaki) daga wannan wuri zuwa wancan*', to indicate walking to move from place to place either for recreation, leisure, exercise, or sports.

**Table 1:** Examples of discrepancies identified during cross-cultural adaptation of IPAQ and their resolution

Issue	Resolution
Selection of vocabulary that responds to the uses and custom in Hausa to avoid ambiguity on the final Hausa version.	Adjustment of the second person pronoun "you" to third person pronouns "he, she" to accommodate gender applicability to Hausa version of IPAQ. In Hausa, the third person pronouns are used for questions because of gender sensitivity. The words "ka" and "ki" are used respectively to distinguish questions for male and female.

Adjustment of English words to match words with familiar concept in Hausa/Nigeria.	Vigorous activity: The word "vigorous" can be misunderstood, as it is not commonly used in Nigeria. The word "very hard" is selected as replacement, because it is more representative of the language used in Nigeria. Moderate activity: the word "hard" was used to replace moderate, because the word is more suitable for the intended activity intensity in Nigeria.

Experiential equivalence: selection of activities equivalent to the Hausa culture.	Item 1: Examples of very hard (vigorous) activities are given that are common in Hausa cultures/Nigeria such as carrying a heavy bucket of water on the head, carrying a two-year-old child on the back and farming (making ridges and hoeing). Item 2: Hard (moderate) activities commonly engaged in Hausa culture/Nigeria are provided as examples, such as pounding grains, sweeping compounds and weeding or planting seedlings. Item 3 and 4: Walking to the farm and work was added as a common walking activity in Nigeria. Sitting doing "course work" was replaced with "house work", and sitting "chatting with families" was included as an example of sedentary activity in Nigeria.

The mean age of the subjects who participated in the psychometric testing was 36.2 ± 9.5 years (range = 20-65 years) and the mean body mass index was 23.9 *± *4.3 kg/m^2 ^(range = 15.4-35.2 kg/m^2^). The participants resting systolic blood pressure, diastolic blood pressure and heart rate were 125.9 ± 19.4 mmHg (range = 90-217), 81.3 ± 10.4 mmHg (range = 60-117) and 77 ± 10.1 beat min^-1 ^(range = 59-109), respectively. The majority were men (n = 56; 54.9%) and married (n = 69; 67.6%), while 29.4% (n = 30) and 40.2% (n = 41) were of the Hausa ethnic group and had university/tertiary education, respectively. A simple majority of the participants were employed (n = 52; 51.0%). Additional characteristics of the sample are presented in Table 2.

**Table 2:** Socio-demographic characteristics of the Participants (N = 102)

Characteristics	n	%
Gender		
Men Women	56 46	54.9 45.1

Marital Status		
Married Single Divorced	69 29 4	67.6 28.4 3.9

Ethnic group		
Hausa Fulani Shuwa Kanuri Others	30 11 9 19 33	29.4 10.8 8.8 18.6 32.4

Religion		
Islam Christianity	81 21	79.4 20.6

Educational level		
Tertiary Secondary Primary Never attended school	41 38 16 7	40.2 37.3 15.7 6.9

Employment status		
Employed Retired Students Housewife Unemployed	52 4 26 9 11	51.0 3.9 25.5 8.8 10.8

Body Mass Index		
Underweight Normal Weight Overweight Obese	8 59 23 12	7.8 57.8 22.5 11.8

Health status		
Excellent Very good Good Fair	22 36 37 7	21.6 35.3 36.3 6.9

### Concurrent validity

Spearman correlation coefficients ranged from moderate (ρ = 0.78) to high (ρ = 0.92), indicating good concurrent validity for the Hausa IPAQ-SF. Total physical activity (MET-min week^-1^) from the Hausa version of IPAQ-SF was significantly and highly correlated with the total physical activity (MET-min week^-1^) from the original English IPAQ-SF (ρ = 0.92, p < 0.001). High positive and significant correlations were also found for time spent (Min week^-1^) in vigorous (ρ = 0.78, p < 0.001), moderate (ρ = 0.86, p < 0.001) and walking (ρ = 0.83, p < 0.001) activities between the Hausa IPAQ-SF and the original English version of IPAQ-SF. The time (Min week^-1^) spent in sitting from the Hausa IPAQ-SF was also significantly (ρ = 0.89, p < 0.001) and positively correlated with the time spent in sitting from the original English version of IPAQ-SF. No meaningful gender and socioeconomic differences were found in the correlation coefficients of all items between the Hausa IPAQ-SF and the original English version (Table 3).

**Table 3:** Concurrent Validity of Hausa IPAQ-SF

Hausa IPAQ vs Original IPAQ	Total (N = 102)	Women (n = 46)	Men (n = 56)
	
	ρ	ρ	ρ
Vigorous PA (Min week^-1^)	0.78**	0.83**	0.77**

Moderate PA (Min week^-1^)	0.86**	0.82**	0.90**

Walking (Min week^-1^)	0.83**	0.81**	0.89**

Total PA (MET-Min week^-1^)	0.92**	0.96**	0.88**

Time spent sitting (Min week^-1^)	0.89**	0.83**	0.78**

ρ - Spearman correlation coefficient between time spent in Physical Activity from the Hausa IPAQ-SF and the original English IPAQ

PA = Physical Activity

Min = Minutes

MET = Metabolic Energy Turnover

**, p < 0.001

**Figure 1:**
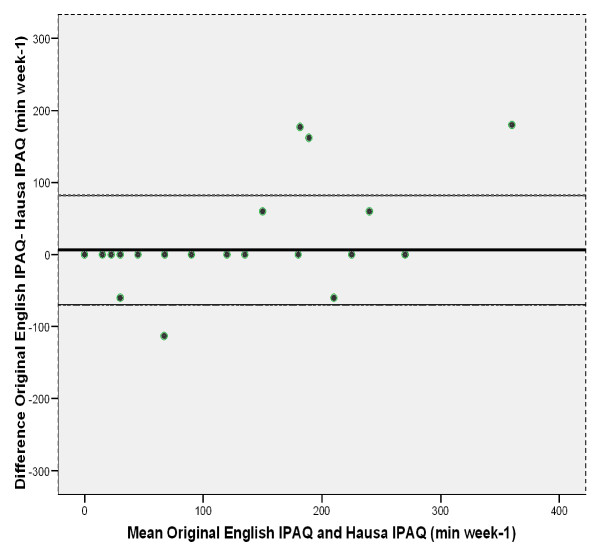
**Bland-Altman plot for time spent in at least moderate physical activity (min week-1) as assessed by the English version of International Physical Activity Questionnaire (IPAQ) and measured using the Hausa version of the IPAQ**. Mean difference: 6.2 min week-1 +/- 2 SD (standard deviations), - 69.8 to 82.1 min week-1 (not significant)

The Bland-Altman plot for the Hausa IPAQ-SF and the English IPAQ demonstrated a small mean difference of 6.19 min week^-1 ^(not significant). However, the 95% limits of agreements were wide, ranging from -69.75 to 82.12 min week^-1^. A few participants that reported more than 50 min week^-1 ^of moderate activity were responsible for the wide range of the 95% limits of agreements (Figure 1).

### Construct validity

Rate Pressure Product (RPP) was only significantly correlated with total time spent in sitting (Min week^-1^) from the Hausa IPAQ-SF (ρ = 0.21, p < 0.01). There was also a weak positive correlation between BMI and time (Min week^-1^) spent in moderate activity (ρ = 0.22, p < 0.04). Rate pressure product and BMI showed no significant relationships with time (Min week^-1^) spent in vigorous and walking activity and total physical activity (MET-min week^-1^) from the Hausa IPAQ-SF (table 4). The men reported higher mean time (Min week^-1^) in vigorous (93.6 vs 62.6, p = 0.10), moderate (101 vs 84.6, p = 0.68), walking (87.1 vs 54.2, p = 0.01) and total physical activity (MET-Min week^-1 ^of 4383.7 vs 3058.1, p = 0.03) than women. However, women spent much more time (Min week^-1^) in sitting activities than men (1773.3 vs 1351.2, p = 0.09). For educational status, participants that completed high school did not report statistical significant higher mean time (Min week^-1^) in doing moderate (98.5 vs 95.8, p = 0.89), walking (75.8 vs 66.3, p = 0.56) and total physical activity (MET-Min week^-1 ^of 3892.3 vs 3417, p = 0.57). Similar those without high school educations did not report statistical significant higher mean time (Min week^-1^) in vigorous (88.5 vs 81.7, p = 0.77) and sitting (1646.9 vs 1363.3, p = 0.34) activities. Participants that were employed did not report statistical significant more time (Min week^-1^) in vigorous (90.6 vs 72.9, p = 0.34), moderate (107.8 vs 87.8, p = 0.20), walking (77.7 vs 70.0, p = 0.56) and total physical activity (MET-Min week^-1 ^of 4008.5 vs 3571.8, p = 0.57) than those who were unemployed, who reported no statistical significant higher time in sitting activity (1664.1 vs 1504.9, p = 0.52) (Not shown in table).

**Table 4:** Construct validity of Hausa IPAQ-SF (N = 102)

Hausa IPAQ	Construct measure	ρ
Vigorous PA (Min week^-1^)	Cardiorespiratory fitness (RPP)	-0.08

Moderate PA (Min week^-1^)	Cardiorespiratory fitness (RPP)	-0.09

Walking (Min week^-1^)	Cardiorespiratory fitness (RPP)	-0.05

Total PA (MET-Min week^-1^)	Cardiorespiratory fitness (RPP)	-0.02

Time spent sitting (Min week^-1^)	Cardiorespiratory fitness (RPP)	0.21**

Vigorous PA (Min week^-1^)	BMI (Kg m^-2^)	-0.16

Moderate PA (Min week^-1^)	BMI (Kg m^-2^)	0.22*

Walking (Min week^-1^)	BMI (Kg m^-2^)	0.05

Total PA (MET-Min week^-1^)	BMI (Kg m^-2^)	0.18

Time spent sitting (Min week^-1^)	BMI (Kg m^-2^)	-0.09

ρ **_**Spearman correlation coefficient between time spent in Physical Activity from the Hausa IPAQ-SF and Cardiorespiratory fitness and BMI

RPP - Rate Pressure Product, BMI - Body Mass Index

PA = Physical Activity

Min = Minutes

MET = Metabolic Energy Turnover

*, p < 0.05; **, p < 0.01

### Reliability

The result of the test-retest reliability is shown in Table 5. Overall, the 1-week ICC ranged from 0.33-0.73, with the lowest value recorded for moderate physical activity and the highest value for vigorous physical activity. The ICC values were significant for all items on the Hausa IPAQ-SF (p < 0.001). Reliability coefficients for vigorous (ICC = 0.82, 95% CI = 0.67-0.91), walking (ICC = 0.63, 95% CI = 0.43-0.77) and total physical activity (ICC = 0.62, 95% CI = 0.43-0.76) were higher in men than in women. The item demonstrating the highest ICC for women was sitting (ICC = 0.67, 95% CI = 0.48-081). Moderate activity resulted in the lowest reliability score for women (ICC = 0.67, 95% CI = 0.48-0.81).

**Table 5:** Test-retest reliability based on intra-class correlation coefficient for the Hausa IPAQ-SF (N = 102)

Hausa IPAQ-SF	Total (N = 102)	Women (n = 46)	Men (n = 56)
	
	ICC (95% CI)	ICC (95% CI)	ICC (95%CI)
Vigorous PA (Min week^-1^)	0.73 (0.55-0.84)	0.44 (-0.19-0.81)	0.82 (0.67-0.91)

Moderate PA (Min week^-1^)	0.33 (0.12-0.51)	0.35 (0.05-0.59)	0.31 (0.01-0.56)

Walking (Min week^-1^)	0.56 (0.39-0.69)	0.43 (0.10-0.67)	0.63 (0.43-0.71)

Total PA (MET-Min week^-1^)	0.61 (0.47-0.72)	0.56 (0.33-0.73)	0.62 (0.43-0.76)

Sitting (Min week^-1^)	0.56 (0.43-0.68)	0.67 (0.48-0.81)	0.40 (0.15-0.60)

ICC = Single measure intra-class correlation coefficient.

PA = Physical Activity

Min = Minutes

MET = Metabolic Energy Turnover

Table 6 shows the results of the socioeconomic status differences in test-retest reliability of the Hausa IPAQ-SF. Intraclass Correlation Coefficients for vigorous (ICC = 0.75, 95% CI = 0.56-0.86), moderate (ICC = 0.36, 95% CI = 0.13-0.55), walking (ICC = 0.57, 95% CI = 0.38-0.71) and total physical activity (ICC = 0.62, 95% CI = 0.46-0.74) were higher among participants with high school educations than in those without high school educations. The reliability coefficient was, however, higher among participants with no high school educations (ICC = 0.64, 95% CI = 0.31-0.83) than in those with high school educations (ICC = 0.53; 95% CI = 0.29-0.70) for sitting activity. While ICC was higher among participants that were employed for vigorous activity (ICC = 0.73, 95% CI = 0.49-0.87) and total physical activity (ICC = 0.65, 95% CI = 0.45-0.78), it was higher for moderate (ICC = 0.40, 95% CI = 0.11-0.63), walking (ICC = 0.60, 95% CI = 0.36-0.76) and sitting (ICC = 0.58, 95% CI = 0.36-0.74) activities among participants that were unemployed.

**Table 6:** Socioeconomic status differences in test-retest reliability of the Hausa IPAQ-SF (N = 102)

Socioeconomic Status	Vigorous PA	Moderate PA	Walking activity	Total PA	Sitting activity
**Educational Qualification**					
High School (n = 79) No High School (n = 23)	0.75 (0.56-0.86) 0.67 (-0.07-0.93)	0.36 (0.13-0.55) 0.23 (-0.24-0.61)	0.57 (0.38-0.71) 0.52 (0.06-0.79)	0.62 (0.46-0.74) 0.57 (0.21-0.79)	0.52 (0.34-0.67) 0.64 (0.31-0.83)

**Employment Category**					
Employed (50) Unemployed (52)	0.73 (0.49-0.87) 0.72 (0.39-0.89)	0.25 (-0.06-0.52) 0.40 (0.11-0.63)	0.51 (0.24-0.71) 0.60 (0.36-0.76)	0.65 (0.45-0.78) 0.56 (0.34-0.72)	0.53 (0.29-0.70) 0.58 (0.36-0.74)

PA = Physical Activity

**Figure 2:**
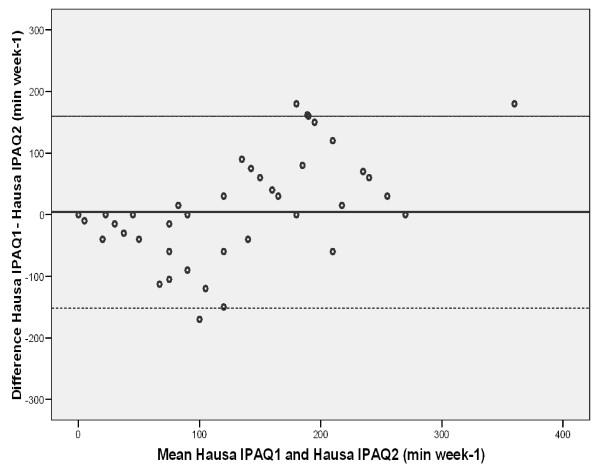
**Bland-Altman plot for moderate activity (min week-1) as assessed by Hausa version of the International Physical Activity (IPAQ) on day 1 and after seven days (test retest reliability)**. Mean difference: 4.3 min week-1 +/- 2 SD (standard deviations), -151.4 to 160.0 min week-1 (not significant)

A Bland-Altman plot for the test-retest of the Hausa IPAQ-SF showed that the mean differences between time spent in moderate activity (min week-^1^) on first and second administrations were small (4.33 min week-^1^) and not significant. However, about ten outliers affected the 95% limits of agreements and the methods were similar (Figure 2).

## Discussion

This study translated and cross-culturally adapted the English IPAQ-SF, and examined aspects of the validity and reliability of the Hausa-IPAQ-SF in Nigeria. The results indicated that Hausa IPAQ-SF had acceptable metric properties for assessing physical activity in healthy adults.

Participants in the pretesting of the Hausa IPAQ-SF misunderstood the question on "walking to travel from place to place" to mean walking to travel out of town rather than for recreation and for transportation to services and destinations as implied on the original English IPAQ-SF. Misunderstanding of the transportation domain item may be attributed to the distinct infrastructural pattern and transportation habit of the African population. Unlike in many countries in North America, Australia and Europe where leisure time physical activity and recreational walking are prevalent [9,25-27], walking is not traditionally associated with recreation in African societies. Active walking may even be erroneously perceived as a sign of being socioeconomically disadvantaged among the urban African elites.

We found higher concurrent validity of the Hausa IPAQ-SF than that reported in a study that directly compared the long and short versions of IPAQ in one language in 12 countries (ρ = 0.83-0.92 vs ρ = 0.51 - 0.64) [6]. Perhaps a better concurrent validity was found in the present study because, unlike in the 12-country study, different languages of only the short IPAQ were compared. However, consistent with previous studies where comparisons of IPAQ data were made with accelerometer monitors [15,28,29], we found higher correlation value for the Hausa IPAQ-SF with vigorous intensity activity than with moderate activity and walking. This finding therefore affirms an earlier report that shows the IPAQ-SF to be more sensitive to intense physical activity than moderate and low intensity activities [12].

In the absence of objective criterion standards for evaluating an absolute estimate of physical activity, the consistency of items on IPAQ with variables known to be related to physical activity such as body mass index (BMI), blood pressure, heart rate, indicators of lipid and glucose metabolism, and fitness index have been used as important validation characteristics [12,13,28], referred to as indirect or construct validity [13,28,30]. Poor construct validity of the Hausa IPAQ-SF in the present study is indicated by a weak, albeit significant, correlation only between rate pressure product as an index of cardiorespiratory fitness index and sitting time (Min week^-1^) (ρ = 0.21). Significant but tenuous correlation between time spent in sitting and rate pressure product implies that higher sitting time as measured on the Hausa IPAQ-SF is related to poor cardiorespiratory fitness (higher RPP indicates lower cardiorespiratory fitness).

The poor relationship between cardiorespiratory fitness and total physical activity, vigorous activity, moderate activity and walking is inconsistent with those of previous studies that reported weak but significant correlations between indices of cardiorespiratory fitness and time spent in vigorous-intensity activity [12,13,31,32], moderate-intensity activity [28] and total physical activity [12,13,28]. Perhaps the contrasting finding between the present study and the previous ones is attributable to the disparate measures of cardiorespiratory fitness utilized. In the present study, RPP as the index of cardiorespiratory fitness incorporated resting heart rate and blood pressure unlike others that utilized submaximal heart rate derived from response to work load as the index of cardiorespiratory fitness [12,13,28]. It has been documented that heart rate response to submaximal workload estimates cardiorespiratory fitness or VO_2_max better than resting heart rate [23].

The reproducibility of the Hausa IPAQ-SF evidenced by substantial agreement for vigorous (ICC = 0.73) and total physical activity (ICC = 0.61), moderate agreement for walking (ICC = 0.56), and sitting activities (ICC = 0.56) and by fair agreement for moderate activity (ICC = 0.33) is similar to those of other studies [12,33]. A study on a Nordic population reported substantial to almost perfect correlations for vigorous activity and sitting, but similar fair correlation (ICC = 0.34) for moderate activity on IPAQ-SF [12]. Another study that assessed the reliability of four physical activity measures including the IPAQ short form in an Australian population found ICC for the questions on moderate activities to be the lowest, ranging from poor to fair agreement (0.16-0.44) [33]. Papathanasiou et al. [34], in a population of Greek young adults found weaker ICC (0.76) for moderate physical activity compared to walking (ICC = 0.78), vigorous (ICC = 0.88) and total physical activity (ICC = 0.92) on the Greek version of IPAQ-SF.

Relatively low reliability for moderate intensity activities compared to vigorous intensity activities as found in the present study may be explained by the low salience nature of moderate activities as previously hypothesized [35]. It is plausible that the time spent in moderate physical activities may be incidental and not easily remembered by participants in the present study. Structured vigorously physical activities like sports and exercise can be more easily recalled compared to some vaguely defined moderate physical activity like "carrying light load" or "multiple household tasks at once" as indicated on the IPAQ-SF [34]. Overall good to acceptable reliability coefficients for all items on the Hausa IPAQ-SF indicate that the instrument is reproducible and internally consistent and is comparable to the findings in previous studies [12,33,34].

Our finding of meaningfully higher reliability coefficient for vigorous intensity activity among men than women is not surprising, because the participants who are men also reported spending more time in all physical activity categories except in sedentary time than the women. Consistently, men have been found to be more physically active than women in a Nigerian population [36]. It is therefore plausible for men in Nigeria to be more consistent in response to items that pertained to intense physical activity than to sedentary activity. Similarly, no statistical significance differences were found in the socioeconomic-based analyses, but participants with higher socioeconomic status as indicated by education (high school education) and employment (those employed) were more physically active than those with low socioeconomic status, and more consistent in their response to questions on vigorous and total physical activity. While it may be difficult to draw any definite conclusion from these findings, they reflect the potential influence of gender and socioeconomic status on physical activity assessment.

Direct comparison of our findings with previous studies should be made with caution, because in the majority of these studies, the accelerometer was utilized as a common objective criterion standard to validate the IPAQ [14,15,28]. In low-income developing countries, the availability and cost of accelerometers remain important research issues [6], and poor receptivity to wearing accelerometers for physical activity assessment has been reported in a sample of urban African adults [5]. Furthermore, the divergent reports on construct validity may suggest that a single measure of aerobic capacity may not be sufficient to determine the construct validity of the physical activity questionnaire. In order to prevent spurious validity of physical activity questionnaires, the selection of comparison instruments that can accurately assess the construct or related dimension measured by the questionnaire has been advocated [37].

Non-utilization of a physical activity diary or any objective measure as a criterion standard to evaluate the validity of the Hausa IPAQ-SF is one limitation of the present study. The choice and availability of appropriate criterion measures are particular issues of concern for the validation of physical activity questionnaires in the low-income countries of Africa [5,38]. In addition, the generalizability of this study may be limited due to the convenience sampling technique utilized and the relative low sample size. Also, the majority of participants were government employees and students with potential higher comprehension and recall ability than may be found in the general population. However, recruitment from diverse workplaces and neighborhoods allowed for a sample with reasonable heterogeneity in age, educational level and ethnic backgrounds.

Despite these limitations, the study demonstrated the feasibility of using the Hausa IPAQ-SF to reliably collect physical activity data in a diverse segment of the Nigerian population. This is the first study in an African country to explore the cultural adaptation and translation of the IPAQ, warranting the need for further validation studies of the Hausa version of IPAQ-SF in other African populations. However, because some of the participants in the present study required assistance to complete the survey, participants in any future national studies in Africa would need to be asked about their ability to independently complete the Hausa IPAQ-SF.

## Conclusion

In conclusion, our results indicate that the Hausa IPAQ-SF has acceptable concurrent validity and test-retest reliability for assessing vigorous intensity, walking, sitting and total physical activity in Nigerian adults. Moderate intensity activity demonstrated almost perfect concurrent validity, but only fair test-retest reliability. The construct validity of Hausa IPAQ-SF provided poor agreement with the rate pressure product and with the BMI, reflecting the need for further assessment in a large sample with objective physical activity and fitness measures, such as the accelerometer and VO_2_Max.

## Competing interests

The authors declare that they have no competing interests.

## Authors' contributions

ALO conceived, designed and provided coordination for the study; conducted the statistical analysis, interpreted the data, and drafted the manuscript. AYO contributed to study design and helped to draft the manuscript. BOA and FOO participated in the design of the study, data acquisition and drafting of the manuscript. HNA, SUA and AAR all contributed to critically revising the drafted manuscript for important intellectual content. All authors read and approved the final manuscript.

## Pre-publication history

The pre-publication history for this paper can be accessed here:

http://www.biomedcentral.com/1471-2288/11/156/prepub

## Supplementary Material

Additional file 1 **Hausa Version of IPAQ-SF**. A final version of the Hausa translated IPAQ-SF and back translated English copy. Click here for file
