# Supplementary material for: The short international physical activity questionnaire: cross-cultural adaptation, validation and reliability of the Hausa language version in Nigeria
Source: BMC Med Res Methodol. 2011 Nov 22;11:156. doi: 10.1186/1471-2288-11-156 (PMC3233518; doi:10.1186/1471-2288-11-156)
Supplement: Additional file 1 — Hausa Version of IPAQ-SF. A final version of the Hausa translated IPAQ-SF and back translated English copy. [file 1471-2288-11-156-S1.DOC]

**TAMBAYOYYIN MOTSA JIKI NA KASA-DA-KASA**

**SALON TAKAITATTU KWANAKI BAKWAI DA SUKA GABATA**

**Don afani akan Matasa da Manya (Shekaru 15-69)**

Tambayoyyin motsa jiki na kasa-da-kasa(wato IPAQ) ta kasu kashi biyu. Akwai Salon Tambayoyyi (guda biyar) masu zurfi,da kuma Salon Takaitattu Tambayoyyi (guda hudu a dunkule). Ana anfani dasu ta hanyar wayar tarho ko sakon hannu-da-hannu. Dalilin wadanna tambayoyyi shi ne samar da ma’auni na baidaya dan samar da alkaluman motsa jiki na kasa-da-kasa akan harkokin da suka danganci motsa jiki da kiwon lafiya.

Salon takaitattu kwanaki bakwai da suka gabata, an tsara su ne musamman domin kula da irin motsa jiki da ke gudana a tsakanin al’umma musamman manya . Tambayoyyin na bukatar sanin tsawon lokaci da aka dauka wajen motsa jiki cikin kwanaki bakwai da suka gabata, a kan harkokin motsa jiki wadannan suke cikin lokacin shakatawa,lokacin aikace-aikacen gida da aikin gona, lokacin aiki da kuma , lokacin zirga-zirga. Abubuwan da ake aunawa su ne: tafiya (tattaki), aiyuka marasa wahala, da kuma aiyuka masu wahala.

TAMBAYOYIN

Ka/ki tuna aiyyukan motsa jiki masu wahala da ka/kika yi cikin kwanaki bakwai da suka gabata. Aiyyukan motsa jiki masu wahala kan bukaci karfi na musamman da kan sanya haki. Ka/ki tuna kawai,da irin aiyyukan motsa jiki da ka/kika yi na tsawon akalla minti goma a kowane lokaci.

1. A kwanaki bakwai da suka gabata, nawa ne adadin kwanakin da ka/kika yi aiyyuka motsa jiki masu wahala kamar su daukar kaya mai nauyi irin su bulo, ko aikin gona irin su tada kunya da kula da kiwon kaji, ko motsa jiki kamar gudu-gudu, wasan bal da tukin keke?

Kwanaki___________________ a sati.

[ ] Ba wani motsa jiki mai wahala.

−−→ A tsallaka zuwa tambaya ta 3

1. Nawa ne adadin tsawon lokacin da ka/kika yi dan yin aiyyuka motsa jiki masu wahala a daya daga cikin wadannan ranaku?

Awa_____________________ A kowace rana

Minti_____________________a kowace rana..

[ ] Ban saniba/ Ban tabbatar ba.

Ka/ki tuna da aiyyukan motsa jiki marasa wahala da ka/kika yi a cikin kwaniki bakwai da suka gabata. Aiyyukan motsa jiki marasa wahala na nufin duk aiyyukan da kan bukaci sa karfi wada kan sanya yin numfashi da yawa fiye da aka saba. Ka/ki tuna kawai da aiyyukan motsa jikida ka/kika yin a tsawon akalla minti goma akan kowane lokaci?

1. A cikin kwanaki bakwai da suka gabata nawo ne aiyyuka motsa jiki marasa wahala kamar daukar kaya marasa nauyi irin su matsar da kujeru, kaya cikin gida, da daukar buhu da yin aikace-aikace cikin gida duk a lokaci daya, ko sare itace da yin sassabe, da yin rawar ibada a coci? Kada a sanya yin tafiya (tattaki).

Kwanaki_____________________a sati.

[ ] Ba wani aikin motsa jiki mara wahala.

−−→ A tsallaka zuwa tambaya ta 5

1. Nawa ne adadin tsawon lokaci da ka/kika yi dan aiyyukan motsa jiki marasa wahala a daya daga cikin wadannan ranaku?

Awa _______________________a kowace rana

Minti_______________________ a kowace rana

[ ] Ban saniba/ Ban tabbatar ba

Ka/ki tuna da adadin lokaci da ka/kika dauka ka/ki na yin tafiya(tattaki) a cikin kwanaki bakwai da suka gabata, wannan ya hada da a gida da wurin aiki, wato yin tafiya(tattaki) daga wannan wuri zuwa wancan, da kuma kowane irin tafiya(tattaki) da ka/kika yi musamman dan shakatawa, wasa, motsa jiki dan dan jin dadi.

1. A cikin kwanaki bakwai da suka gabata nawa ne adadin kwanaki da ka/kika yi ka/ki na tafiya(tattaki) akalla na minti goma a lokaci daya?

Kwanaki_____________________ a sati

[ ] Ba wani tafiya (tattaki)

−−→a tsallaka zuwa tamboya ta 7

1. Nawa ne adadin tsawon lokaci da ka/kika yina tafiya (tattaki) a daya daga cikin wadanna ranaku?

Awa ________________________ a kowace rana

Minti________________________ a kowace rana

[ ] Ban saniba/ Ban tabbatar ba.

Tamboya ta karshe ta shafi tsawon lokaci da ka/kika dauka dan zama a zaune a ranakun aiki a cikin kwanaki bakwai da suka gabata. Wannan ya hada harda lokaci da ka/kika dauka a wurin aiki da gida, yayin shakatawa. Wannan na iya kunsar lokaci da aka dauka ana zaune a ofis, ziyartar abokai, zama dan yin karatu ko kishingida, dan kallon talabijin?

1. A cikin kwanaki bakwai da suka gabata, nawane adadin tsawon lokaci da ka/kika dauka a zaune a ranakun aiki?

Awa_______________________ a kowane rana.

Minti_______________________ a kowane rana.

[ ] Ban saniba/ Ban tabbatar ba.

**BACK TRANSLATED ENGLISH VERSION**

**INTERNATIONAL PHYSICAL ACTIVITY QUESTIONNAIRE**

**SHORT LAST 7 DAY VERSION**

**For use with Young and Middle- age Adults (15-69 years)**

The International Physical Activity Questionnaire (IPAQ) comprises a set of 4 questionnaire. Long (5 activity domains asked independently) and short (4 generic items) versions for use by either telephone or self-administered methods are available. The purpose of the questionnaires is to provide common instruments that can be used to obtain internationally comparable data on health- related physical activity.

IPAQ short (last 7 day) version was designed primarily for population surveillance of physical activity among adults. It asked about the time you spent being physically in the last 7 days on three specific types of activity undertaken in the four domains of leisure time, domestic and gardening, works, and transports. The specific types of activity that are assessed are walking, hard activities and very hard activities.

QUESTIONS

Think about all the **very hard** activities that you did in the **last 7 days. Very hard** activities take strenuous physical effort and make you breathe much harder than normal. Think only about those physical activities that you did for at least 10 minutes at a time.

1. During the **last 7 days**, on how many days did you do **very hard** physical activities like carrying heavy load such as blocks, bucket of water on the head or a two years old child on the back; farming such as making ridges, hoeing in farm or poultry works; or doing exercises such as jogging, playing football and riding bicycle?

_______**days per week**

**[ ]** No vigorous physical activities → **Skip to question 3**

2. How much time did you usually spend doing **very hard** physical activities on one of those days?

_______**hours per day**

**_______minutes per day**

**[ ]** Don’t know/Not sure

Think about all the **hard** activities that you did in the **last 7 days**. **Hard** activities refer to activities that take some physical effort and make you breathe a little harder than normal. Think only about those physical activities that you did for at least 10 minute at a time.

3. During the **last 7 days**, how many days did you do **hard** physical activities like carrying light loads such as moving household items, furniture and carrying sack; sweeping the compound; pounding grains; doing multiple household task all at once; weeding or planting seedlings; and spiritual dancing in church? **Please do not include walking**.

_____**days per week**

**[ ]**  No moderate physical activities → **Skip to question 5**

4. How much time did you usually spend doing **hard** physical activities on one of those days?

_______**hours per day**

**_______minutes per day**

**[ ] Don’t know/Not sure**

Think about the time you spent **walking** in the **last 7 days.** This includes at work and at home, walking to move from place to place, and any other walking that you might do solely for recreation, sport, exercise, or leisure and to move to farm or work.

5. During the **last 7 days,** on how many days did you **walk** for at least 10 minutes at a time?

______**days per week**

**[ ] No walking** → **Skip to question 7**

6. How much time did you usually spend **walking** on one of those days?

________**hours per day**

**________minutes per day**

**[ ] Don’t know/Not sure**

The last question is about the time you spent **sitting** on week days during the **last 7 days.** Include time spent at work, at home, while doing house work and during leisure time. This may include time spent sitting at a desk, visiting friends, chatting with family, reading, or sitting or lying down to watch television.

7. During the **last 7 days,** how much time did you spend **sitting** on a **week day?**

_______**hours per day**

**_______minutes per day**

**[ ] Don’t know/Not sure**
